# Enabling equitable open access to commercially funded research publications

**DOI:** 10.1371/journal.pmed.1005207

**Published:** 2026-08-27

**Authors:** Anja Schmidt, Christopher C. Winchester, Catherine Skobe, Diane R. Stothard, Eric Topol

**Affiliations:** 1 Takeda Development Center Americas, Inc., Cambridge, Massachusetts, United States of America; 2 Oxford PharmaGenesis, Oxford, United Kingdom; 3 Pfizer, New York, New York, United States of America; 4 Eli Lilly and Company, Indianapolis, Indiana, United States of America; 5 Scripps Research, La Jolla, California, United States of America

## Abstract

In this Perspective, Christopher Winchester and colleagues discuss how open access options for publishing commercially-funded research are often restricted, examine the consequences of this inequity, and argue for funder-neutral publishing policies.

## Teaser

Pharmaceutical companies fund much biomedical research, yet many journals restrict open access publishing options for peer-reviewed articles reporting commercially funded research. Expanded publishing options, such as funder-neutral publishing policies, will help address this inequity.

Pharmaceutical companies fund a substantial proportion of the biomedical research that directly affects disease understanding, treatment, and public health [1]. However, the peer-reviewed publications of these findings are often not freely available, because many journals limit or deny open access for commercially funded research. We argue that there is an ethical obligation to make clinical research conducted in humans freely and universally accessible to the public—however it is funded—through equitable open access to the peer-reviewed version of record.

Access underpins public trust in science, and The World Medical Association Declaration of Helsinki recognizes that conducting research involving human participants carries an ethical duty to make the results publicly available [2]. Indeed, the World Health Organization is pursuing universal access to reliable healthcare information in collaboration with Healthcare Information For All [3]. In addition, the UNESCO Recommendation on Open Science [4] encourages openness, transparency and collaboration, including sharing of knowledge, methods and data to improve the rigor, reliability, impartiality and societal impact of science. Sharing research methods and results advances science and healthcare as global public goods, yet much information remains behind the paywalls of reputable peer-reviewed academic journals.

Paywalls may be minor budgetary issues for well-funded institutions but can create significant barriers to healthcare professionals, researchers, guideline developers and other stakeholders, especially those outside academic institutions, those in low- and middle-income countries and those in community settings. The power of evidence-based medicine relies on evidence availability, especially in an information system increasingly dependent on artificial intelligence. Healthcare professionals without access to institutional subscriptions may be unable to access the full-text research articles needed to inform their patient care, and restricted access to the literature base can limit the ability of healthcare professionals, guideline developers and policymakers to evaluate, validate and synthesize the full breadth of available evidence [5].

Patients, caregivers and the public can also lack free and easy access to medical research findings that directly affect their lives [6]. Clinical trial volunteers may not have access to peer-reviewed publications of the trial results; access to peer-reviewed publications and ideally to accompanying plain language summaries could enhance patient understanding of treatment options and public trust in healthcare. Barriers to accessing reliable, peer-reviewed evidence mean people may turn to potentially less reliable and/or less relevant sources of information and could be exposed to misinformation [7].

Pharmaceutical company research is part of a complex scientific and medical research and development ecosystem [8]. Regardless of how research is funded, shouldn’t the published findings from studies involving human volunteers be freely accessible to everyone, everywhere in the world? Is it appropriate that journal publishers, which are (with the exception of nonprofit publishers such as PLOS) also commercial entities, determine which research is freely available?

Pharmaceutical companies are following public research funders in publishing open access. As part of their commitments, many pharmaceutical companies publish all medically important trial results in reputable peer-reviewed journals [9,10]. But many high-impact journals do not offer commercial funders the same open access options as those offered to noncommercial research funders [11] despite pharmaceutical companies’ willingness to cover the associated fees. Some journals allow ’green’ open access (i.e., self-archiving) to the accepted author manuscript but paywall the published official peer-reviewed version of record. Although preprints provide a mechanism for early sharing of research findings, they are not a substitute for the peer-reviewed version of record, which remains essential for validated scientific communication and inclusion in clinical and policy decision-making. While journals sometimes provide free-to-read access to key articles relevant to global public health, this falls short of universal open access to clinical study publications in trusted journals.

Why is universal open access to commercially funded research publications unrealized? The Open Science movement is strongly rooted in a not-for-profit ethos, and there has historically been skepticism towards for-profit research, sometimes justifiably so, owing to documented cases in which commercial interest influenced study design, data interpretation, publication practices and research independence in commercially funded research [12]. Nevertheless, potential conflicts of interest do not inherently compromise research integrity, and integrity concerns are mitigated through appropriate disclosures, rigorous peer review, data sharing and independent scrutiny of research outputs. Research from pharmaceutical companies now attains high standards of integrity and reporting, including publication in reputable peer-reviewed journals [9,10]. Readers should always consider who has funded the research during their appraisal of outputs. However, the funding source should not determine whether or not research findings are freely and universally accessible. If rigorous, ethical and transparently reported, research should be judged on its merits rather than the funding source alone.

Academic publishing is a multi-billion-dollar industry dominated by a small number of major players [13]. The success of noncommercial funders in requiring open access publication may paradoxically pressure journals to protect subscription fees by continuing to paywall commercially funded research. This often means that basic research is freely accessible, but research most relevant to healthcare is not. If publishers are gatekeepers of the scientific robustness of commercially funded research, then this is incompatible with their commercializing the resulting publications. Ethically, all scientific research findings should be universally accessible regardless of funding: the fruits of our shared human endeavor should be available to anyone who needs them, anywhere in the world.

New publication models are needed to ensure that authors of commercially funded research have the same rights as other authors to publish open access in respected, high-quality, peer-reviewed journals. These models must sustain a healthy publishing ecosystem by upholding rigorous peer review standards and guarding against predatory publishing. Transformative agreements—which have been successfully used by academic institutions to combine subscription fees and article processing charges—could allow pharmaceutical companies to expand open access publishing without restricting journal choice for authors. The key issue remains that some journals do not allow open access for company-funded research publications, even when a fee is paid, despite the companies’ desire to make their research outputs available and universally accessible.

Creativity is needed for a healthy future publishing ecosystem that meets all stakeholders’ needs. We call on pharmaceutical companies and publishers to work together towards equitable, worldwide open access to all peer-reviewed and published medical research findings for the sake of transparency, for the mission of collaborative scientific dialogue and for public health benefit. Specifically, we implore publishers to introduce funder-neutral open access policies and clearer positions on self-archiving of the version of record (i.e., green open access) to ensure all company-funded clinical research is free to read and reuse from the date of publication [14].
